# Classification and visualization of neural patterns using subspace analysis statistical methods

**DOI:** 10.1186/1471-2202-13-S1-P74

**Published:** 2012-07-16

**Authors:** Jun Xia, Marius Osan, Emilia Titan, Riana Nicolae, Remus Osan

**Affiliations:** 1 Department of Mathematics and Statistics, Georgia State University, Atlanta, GA, 30303, USA; 2 Statistics and Econometrics Department, Economic Cybernetics, Statistics and Informatics Faculty, Academy of Economic Studies, Bucharest, Romania; 3 Management and Marketing Faculty, Artifex University, Bucharest , Romania; 4 Neuroscience Institute, Georgia State University, Georgia State University, Atlanta, GA, 30303, USA

## 

The size and complexity of neural data is increasing at a dramatic pace due to rapid advances in experimental technologies. As a result, the data analysis techniques are shifting their focus from single-units to neural populations. The goal is to investigate complex temporal and spatial patterns, as well as to present the results in an intuitive way, allowing for detection and monitoring of relevant neural patterns.

As an example of neural recordings, we first look at large-scale recordings in the hippocampus, a brain region that plays a crucial in converting short-term memory into long-term memory (memory consolidation). Here, experimental constraints may require a small number of trial repetitions, preventing the acquisition of comprehensive statistics commensurate with the large number of units recorded. This under-sampling of the population dynamics poses challenges for the direct application of complexity-reduction techniques, such as Multiple Discriminant Analysis (MDA), a subspace projection (eigenvalues/eigenvector) statistical method. However, upon solving these types of issues through regularization techniques, these methods can be used along with other unsupervised subspace projection methods, (e. g. Principal Component Analysis (PCA)), or data-mining techniques, to facilitate the understanding and monitoring of the dynamics of these neural populations, reflecting the network-level ensemble representations [1]. Application of these methods can be used to differentiate between somato-sensory and memory components of the hippocampal representations [2]. Using hierarchical clustering, we determined that the recorded neural population can be divided into two classes: units that respond in an invariant fashion to the stimuli of all intensities and units that modulate their responses as a function of the magnitude of the stimuli. More importantly, our results suggest that the neurons belonging to the first category (invariant responses), are the main drive for the reactivation of the memory traces, in contrast to the other type of units.

These techniques can be used directly on other types of neural patterns, such as the ones obtained using optical imaging data. On these data sets, we are investigating how dynamics of odor responses in the primary receptor neurons of awake rats are shaped by the temporal features of the active odor sniffing. Our analyses indicate that the dynamics of neural representations depend non-linearly on odor identity and concentration, as well as breathing rhythms of the rats. In addition, we examined how a recent visualization technique for multi-electrode spike trains [3], which relies on an implementation of the Kohonen self-organizing maps, performs on our neural data. In particular, we looked at the correlation between the events detected by the subspace analysis (supervised method) and the ones detected by the visualization technique (unsupervised method).
